# Cost-effectiveness analysis design for interventions to prevent children's oral disease

**DOI:** 10.3389/froh.2024.1428638

**Published:** 2024-07-18

**Authors:** Joanne Spetz, Johnie Rose, James G. Kahn, Tracy Lin, Douglas Levy, Oksana Pugach, Susan Hyde, Belinda Borrelli, Michelle Henshaw, Molly Martin, Suchitra Nelson, Francisco Ramos-Gomez, Stuart A. Gansky

**Affiliations:** ^1^ Philip R. Lee Institute for Health Policy Studies and Healthforce Center, University of California, San Francisco, CA, United States; ^2^ Center for Community Health Integration, Case Western Reserve University School of Medicine, Cleveland, OH, United States; ^3^ Institute for Health and Aging, School of Nursing, University of California, San Francisco, CA, United States; ^4^ Mongan Institute Health Policy Research Center, Massachusetts General Hospital and Harvard Medical School, Boston, MA, United States; ^5^ Institute for Health Research and Policy, University of Illinois Chicago, Chicago, IL, United States; ^6^ Center to Address Disparities in Children's Oral Health, School of Dentistry, University of California, San Francisco, CA, United States; ^7^ Goldman School of Dental Medicine, Boston University, Boston, MA, United States; ^8^ College of Medicine, University of Illinois, Chicago, IL, United States; ^9^ School of Dental Medicine, Case Western Reserve University, Cleveland, OH, United States; ^10^ Section on Pediatric Dentistry, Center for Children's Oral Health, School of Dentistry, University of California, Los Angeles, CA, United States

**Keywords:** cost-effectiveness analysis, pediatric, oral disease, children, economic evaluation

## Abstract

**Introduction:**

In 2015, the National Institute of Dental and Craniofacial Research (NIDCR) launched the Multidisciplinary Collaborative Research Consortium to Reduce Oral Health Disparities in Children, supporting four randomized trials testing strategies to improve preventive care. A Coordinating Center provides scientific expertise, data acquisition and quality assurance services, safety monitoring, and final analysis-ready datasets. This paper describes the trials' economic analysis strategies, placing these strategies within the broader context of contemporary economic analysis methods.

**Methods:**

The Coordinating Center established a Cost Collaborative Working Group to share information from the four trials about the components of their economic analyses. Study teams indicated data sources for their economic analysis using a set of structured tables. The Group meets regularly to share progress, discuss challenges, and coordinate analytic approaches.

**Results:**

All four trials will calculate incremental cost-effectiveness ratios; two will also conduct cost-utility analyses using proxy diseases to estimate health state utilities. Each trial will consider at least two perspectives. Key process measures include dental services provided to child participants. The non-preference-weighted Early Childhood Oral Health Impact Scale (ECOHIS) will measure oral health-related quality of life. All trials are measuring training, implementation, personnel and supervision, service, supplies, and equipment costs.

**Conclusions:**

Consistent with best practices, all four trials have integrated economic analysis during their planning stages. This effort is critical since poor quality or absence of essential data can limit retrospective analysis. Integrating economic analysis into oral health preventive intervention research can provide guidance to clinicians and practices, payers, and policymakers.

## Introduction

A growing body of research incorporates economic analysis into studies of oral health prevention and treatment programs. Incorporating cost and preference outcomes provides important information to improve practice and policy (1). Previous work has examined the cost-effectiveness of preventive oral health practices including water fluoridation; application of sealants, topical fluoride, and fluoride varnish; and tooth brushing with fluoride toothpaste. The cost-effectiveness of water fluoridation, tooth brushing, fluoride varnish, dental sealants, and preventive care provided in pediatrician offices is now well-accepted (2–10). The challenges associated with economic studies are illustrated by a recent retrospective analysis reporting that prior research on water fluoridation overstated its benefits (11). There is comparatively sparse literature on the cost-effectiveness of treatment for dental disease, with most studies describing only costs (2, 12).

In 2015, the National Institute of Dental and Craniofacial Research (NIDCR), a component of the United States National Institutes of Health, launched the Multidisciplinary Collaborative Research Consortium to Reduce Oral Health Disparities in Children (MCRC OHDC). Through this initiative, NIDCR provided funding for studies focused on promoting oral health and preventing and managing dental disease for children facing health disparities (13). Four NIDCR-funded project teams are implementing innovative randomized trials that are rigorously testing strategies to improve preventive care. All four behavioral theory-driven trials include cost data collection and economic analysis plans.

The MCRC OHDC studies include a test of different financial incentives to families to adopt early childhood caries prevention habits (BEhavioral EConomics for Oral Health iNnovation Trial: BEECON; clinicaltrials.gov registration number NCT03576326); a family-focused community health worker program [Coordinated Oral Health Promotion (CO-OP) Chicago; NCT03397589]; an interactive and customized parent-targeted text message program to improve oral health in children attending urban pediatric clinics (Interactive Short Messages Initiate Lasting Education, iSmile; NCT03294590) (14); and a bundled practice-and provider-level intervention in primary care that includes theory-based education on oral health, referral to dental care, and integration of oral health assessment into the electronic health record (Providers Against Cavities in Children's Teeth: PACT; NCT03385629). These studies started with a multi-year planning and feasibility piloting phase. After meeting their *a priori* milestones and undergoing external scientific committee and NIDCR review, the studies were approved to continue to full prevention trials. Each study developed a tailored approach to its intervention, outcome measures, and cost measures, with a single Coordinating Center (CC) providing collaborative scientific and clinical trials expertise, data acquisition and quality assurance services, project and participant safety monitoring, and final analysis-ready data sets. The CC also fosters the use of conceptually similar measures across study teams and supports the development of de-identified public-use data sets.

This article describes the economic analysis strategies of all four trials, placing these strategies within the broader context of contemporary economic analysis methods. The range of potential economic analysis approaches available and rationales for the selection of specific methods for each study are described, illustrating the practical challenges of conducting economic analysis in oral health clinical trials.

## Methods

As the four studies commenced, the CC established a Cost Collaborative Working Group (CCWG), which developed a form for study teams to share information on the components of their economic analyses. These components were (1) the perspective of the analysis, which identifies the parties to be considered in the measurement of costs and benefits; (2) benefit measures used, including process, health outcomes, and quality of life; and (3) cost measures including costs to dental and medical practices, costs to payers, costs to participants, and research costs.

Of the several different approaches to economic analysis of health care programs, two of the most common are being used in the MCRC OHDC programs: cost-effectiveness analysis and cost-utility analysis (15). Cost-effectiveness analysis (CEA) measures the incremental costs of alternatives relative to incremental benefits, with benefits measured using a single consistent effectiveness metric, such as decrease in number of patients with caries or number of additional applications of fluoride varnish. Cost-utility analysis (CUA) transforms the benefits of the alternatives into a measure of the quantity and quality of life, allowing comparisons across heterogeneous programs/technologies. The quality of life for each health state experienced is based on individual preferences and is standardized to a 0–1 scale, where 1 is generally defined as perfect health and 0 is death. The preference values, or “weights”, are multiplied by the duration in which a person experiences each health state, yielding life expectancy estimates weighted by the burden of disease. The two most common preference-weighted measures are quality-adjusted life years (QALYs) and disability-adjusted life years (DALYs). QALYs adjust for health state utilities, while DALYs adjust for the degree of disability experienced.

For these analyses, the costs and benefits of each alternative are measured, and results are typically presented as the ratio of the incremental cost (i.e., cost of alternative B - cost of alternative A) to the incremental benefit (i.e., benefit of alternative B - benefit of alternative A) of moving from A to B. When there are multiple alternatives, one is selected as the baseline against which the others are compared; the baseline is typically the current standard of care. This ratio is the incremental cost-effectiveness ratio (ICER) (16).

The decision on perspectives of economic analyses is an important one and is typically tied to the study research questions and the stakeholders of interest—with many studies considering more than one perspective. Whose costs and whose outcomes should be considered? The perspective could be that of the individual and/or their family, the dental clinic or practice, the payer (often Medicaid), or society as a whole (17). The Second Panel on Cost-Effectiveness recommended that the perspectives of society and of the health care sector be reported in all studies (18).

Utilizing good research practices for CEA (19), study teams indicated the data sources for each component of the economic analysis that could be collected for their study. As each study refined its program and data collection protocols, tables summarizing the economic data components were updated. CCWG members met regularly via conference call to discuss their progress in developing their data collection processes and measures, share best practices and standardize measures, and discuss solutions to emergent challenges.

## Results

### Framework, perspective and time horizon of the analyses

All four trials will calculate ICERs; the BEECON and PACT studies also will conduct CUA. The data available and the stakeholders of interest for each trial shaped the perspective and time horizon of each economic analysis (Table 1).

**Table 1:** Perspective and time horizon of MCRC OHDC studies.

Study	Type of economic analysis	Perspective/Viewpoint	Time horizon
BEECON	Cost-effectiveness	Family	1 year
	Cost-utility	Medicaid	2 and 5 years
		Societal	10 and 20 years
CO-OP	Cost-effectiveness	Health clinics	One year
		Social service agencies	
iSmile	Cost-effectiveness	Health clinics	Start-up and steady state
		Payers (Medicaid & private)	
PACT	Cost-effectiveness	Individual/family	2 and 5 years
	Cost-utility	Health clinics	
		Medicaid	
		Societal	

BEECON offers financial incentives to low-income families to engage in oral health prevention activities. The first logical viewpoint is that of the family, because family members decide whether to engage in prevention activities based on personal assessments of costs and benefits. Over the long-term, whether the project is cost-effective or cost-saving from the viewpoint of Medicaid, which is the primary payer of dental care for the target population, may provide guidance regarding whether Medicaid should offer financial incentives for prevention activities as part of dental insurance. Note that families do not have any reason to be concerned about the costs borne by Medicaid, and Medicaid does not have financial incentive to be concerned about the costs borne by families; thus, the analyses for each of these viewpoints might reach different conclusions. For this reason, BEECON also will consider the societal perspective, combining all costs and all benefits, regardless of whether they accrue to families or to Medicaid.

CO-OP is engaging community health workers to improve preventive care among enrolled families. Quantifying family-level costs is beyond the scope of CO-OP. Because community health workers are both clinic-based and community-based in the CO-OP model, the analysis will instead consider the perspective of pediatric medical clinics and social service agencies; if the program is cost-effective from their perspective, these stakeholders may choose to employ community health workers permanently.

iSmile is recruiting families from urban pediatric clinics in low-income areas and assessing an intervention consisting of sending automated, interactive, and customized text messages to parents of children under the age of seven about oral health. iSmile is examining the incremental costs of starting and maintaining this intervention from the perspectives of the organizations that might purchase such a technology, specifically pediatric medical practices and federally qualified clinics, as well as payers.

Finally, PACT is measuring the economic impact of each multi-level intervention (practice and provider) on costs of care from the societal perspective, which will be examined over a two-year follow-up period. Costs associated with utilization will be divided into direct (dental and non-dental) and indirect dental costs. Information on these *direct dental* costs will be obtained using Medicaid claims data. *Non-dental direct* costs such as transportation to/from dental visits, and *dental indirect* costs (i.e., the value of lost productivity or leisure time related to receiving care or to dental morbidity) will be elicited using a resource use data collection instrument such as the Annotated Cost Questionnaire (20).

The time horizon for analysis is often determined by data availability. Assessments about the potential for cost savings that may be accrued by health care providers or payers may be adequate with short-term data, and stakeholders may prefer to focus on relatively short time horizons. In contrast, CUA are often calculated over the course of the lifetimes of the individuals affected by a program. BEECON's CUA will consider a 1-year time period to compute a short-term ICER, as well as 2- and 5-year time periods to compute mid-term ICERs, and 10- and 20-year time periods to compute long-term ICERs. CO-OP swill track data over a 1-year intervention period to compute ICERs that cover one year. iSmile will split costs into start-up costs and steady state costs so that stakeholders will be able to assess both short-term and ongoing resource requirements. These data will be mapped to benefits accruing over time to estimate ICERs at any time interval of interest. PACT's CUA will use a 5-year time horizon. Because widely-accepted rule-of-thumb thresholds regarding whether interventions are deemed cost-effective, such as $50,000–$150,000 per QALY, are based on lifetime time horizons and societal perspective analyses (21, 22), caution must be exercised when using these benchmarks to draw conclusions about whether programs assessed with shorter time horizons or from other perspectives are valuable.

### Measurements of benefits

Measuring benefits of a health care program involve examining process measures, health outcomes measures, and financial benefits. For all four MCRC OHDC programs, a key process measure is the level of participation of individuals in the study (Table 2). The dental services provided to child participants are another category of process measure. All four trials include data on preventive services received by study participants, obtained from several different sources. BEECON's measurement of preventive care is a measure of completed preventive dental visits from the Early Head Start (EHS) electronic participant record system. BEECON is obtaining child dental prevention information from Bluetooth toothbrushes. CO-OP, iSmile and PACT will use a questionnaire completed by children's parents/caregivers to obtain information about preventive services. CO-OP and iSmile also measure oral health behavior change (e.g., reduction in sugar-sweetened beverages and cariogenic foods) and PACT will use Medicaid claims data. CO-OP will supplement caregiver-reported data with data on preventive services referred by community health workers.

**Table 2:** Measurement of benefits in MCRC OHDC studies.

Study	Process measures	Outcome measures
BEECON	Number of participants	Toothbrushing frequency
	Syncing frequency for toothbrush	Plaque score
		Annual dental visit
		Additional dentist visits
		Quality of Life (ECOHIS)
		Program satisfaction
		QALYs
		Staff satisfaction
CO-OP	Number of participants	Toothbrushing frequency
	Number of educational sessions provided	Emergency Department visits
	Number of preventive services provided	Plaque score
		Sugar-sweetened beverage consumption
		Quality of Life (ECOHIS)
		Program satisfaction
		Staff satisfaction
iSmile	Number of participants	Caries incidence
	Text messages received/responded to	Lost teeth
	Oral health behaviors	Abscesses
	Changes in theory-based mediators (motivation, self-efficacy, outcome expectations)	Oral health behaviors (toothbrushing, diet, sugar sweetened beverages, use of fluoride toothpaste, preventive dental visits)
		Quality of Life (ECOHIS)
		Program satisfaction
		Parent's own oral health behavior
PACT	Number of participants	Caries experience
	Number of preventive services provided	Caries treatment
	Number of sealants applied	Emergency department visits
		Quality of life (ECOHIS)
		Program satisfaction
		QALYs

All four trials will use parent surveys to measure participant satisfaction with the program. BEECON will conduct satisfaction surveys of EHS staff and use three measures from the Basic Screening Survey of the Association of State and Territorial Dental Directors (23): any untreated caries (dt >0), any caries experience (dmft >0), and severe caries (dmft ≥4). CO-OP will obtain data on emergency department visits and hospitalizations through a parent questionnaire, which also will provide information about whether child participant consumption of sugar-sweetened beverages declines.

iSmile and PACT will examine a common set of health outcomes measured through clinical oral health examinations: caries severity (number of decayed, missing, or filled primary teeth known as dmft), number of carious lesions (ds), number of subjects with caries, and number of primary teeth lost due to caries (mt); incremental effectiveness will be operationalized as between-arm differences in each measure. iSmile will use estimates from the literature as well as estimates from publicly available data sources to project downstream benefits of adverse outcomes avoided. PACT will use Medicaid claims data to measure caries treatment, along with numbers of dental-related emergency department visits and hospitalizations.

BEECON and PACT will conduct a CUA. To circumvent the lack of health state utility data relevant to pediatric oral health both teams are using the non-preference-weighted Early Childhood Oral Health Impact Scale (ECOHIS), a widely-adopted and well-validated quality-of-life instrument (24–26), to assess quality-of-life outcomes. Data for the ECOHIS scores can be derived from dental records and Medicaid claims. As of yet, there is no analysis mapping ECOHIS scores to preference weights. Accordingly, for the purpose of calculating QALYs for CUA, both studies will apply utilities from diseases similar in nature and severity to pediatric caries and determine the best utilities to use as proxies for caries. The two studies are first mapping out the utilities from diseases similar to caries in children; the result is a range of utilities from the least severe to most severe proxy disease. One example of such a disease is otitis media, which is a common proxy disease utilized by the United Kingdom National Institute for Healthcare Excellence to calculate cost-effectiveness of caries prevention interventions (27). Next, PACT is using a validated scoring system that relates dmf index scores to probability of symptom outcomes (28) to translate prevalence data based on dmf scores to the prevalence of different symptom levels stemming from caries. This process generates distinct utilities for different caries severity that can be employed in the CUA.

### Measurements of costs

All four trials are tracking the costs of their programs and study-specific costs (Table 3), measuring training costs, implementation costs, personnel and supervision costs, service costs, and supplies and equipment costs using project records. BEECON, iSmile, and PACT are tracking the cost of dental services provided. Sources of these data include staff timesheets, direct observation (e.g., time motion tracking), mileage reimbursement requests, contracts, and clinic financial data. PACT is using Medicaid claims data to measure the cost of dental services. They will also measure indirect costs associated with travel and lost work and leisure time from attending dental visits, using an adaptation of the Annotated Cost Questionnaire (20).

**Table 3:** Measurement of costs in MCRC OHDC studies.

Study	Research-specific costs	Program costs	Program financial savings
BEECON	Research team personnel	Personnel time and supervision	No savings measurement / evaluation planned
	Development and implementation of research plan	Training and implementation	
	Participant incentives	Supplies and equipment	
		Materials development	
		Mileage	
		Telephone	
		Dental services	
CO-OP	Research team personnel	Personnel time and supervision	No savings measurement / evaluation planned
	Development and implementation of research plan	Training and implementation	
	Participant incentives	Supplies and equipment	
		Materials development	
		Mileage	
		Telephone	
iSmile	Research team personnel	Personnel time and supervision	Savings from reduced cost of caries treatment
	Development and implementation of research plan	Training and implementation	
	Participant incentives	Supplies and equipment maintenance	
		Text-messaging development and maintenance	
		Dental services	
PACT	Research team personnel	Personnel time and supervision	Savings from (reduced) cost of caries treatment
	Development and implementation of research plan	Training and implementation	Savings from saved work days
	Participant incentives	Supplies and equipment	Savings from avoided emergency department visits
		Materials development	
		Mileage	
		Telephone	
		Dental services	
		Costs to participants (travel, lost work/leisure time)	

All these trials are making clear distinctions between research costs and program costs, including only program costs in the formal CEA.

## Discussion

Consistent with best practices, the four trials described here have integrated economic analyses beginning at the planning stages of each trial to prevent absence of essential data for analysis (17, 19). Data are being collected from electronic dental and medical records, Medicaid claims, direct dental examination, participant and staff questionnaires, parent questionnaires, and project records. In addition, some of the trials are conducting unique analyses to support their economic analysis. For example, CO-OP is measuring program costs by recording the time spent by community health workers. Similarly, BEECON and iSmile are relying on estimates of program costs obtained from time-and-motion studies, which cannot be done retrospectively. PACT is obtaining information about the costs borne by participants through questionnaires; without integrating an economic evaluation component from the beginning of the trial, the questionnaires likely would have focused only on participant satisfaction and provided no estimate of the time required to participate.

All four trials are conducting a CEA, with the BEECON and PACT studies also developing QALY measures for a CUA, which is challenging due to the potential burden of obtaining valid data that can be used to determine QALYs. Including direct elicitation methods such as time-trade off or standard gamble, or including a preference-based health state classification system, such as Child Health Utility 9D (CHU-9D) or Health Utilities Index 2 (HUI2) (29), was not feasible for the MCRC OHDC studies due to the additional resource needs and subject burden inherent to gathering such data. In addition, significant concerns exist that generic preference-based measures are not as sensitive to changes in relevant health domains as disease-specific quality of life measurement instruments are (30, 31).

Although all four trials are assessing whether their preventive interventions might produce cost savings for clinics/practices and payers/Medicaid, none are applying a strict method of cost-benefit analysis (CBA), in which all benefits are converted to monetary values and net costs (or net savings) are calculated. In dental research, CBA is uncommon in part because willingness-to-pay measures are not readily available to translate health and other intangible benefits into dollar values, and willingness-to-pay may vary across settings (32). Future research should aim to develop better and more flexible measures of both utilities and willingness-to-pay measures that can support comprehensive CUA and CBA. A more comprehensive understanding of the relative benefits of interventions to prevent pediatric caries would also consider the long-term (lifetime) impact of improved oral health behaviors and oral health. Together such efforts would help advance more comprehensive and strategic planning in dental public health.

## Conclusion

Integrating economic analysis into research on the efficacy of oral health preventive interventions can provide important guidance to dental clinics and practices, payers, and policymakers in oral health policy and practice. Economic data often can be easily compiled as part of the research protocol, but some data is best collected prospectively using dedicated instruments and methods, so proper planning is essential.

Each trial in the MCRC OHDC Consortium was developed based on behavioral science theory delineating how each intervention can affect oral health outcomes, and the economic components of each were integrated as part of the design phase. There are some common data elements across the studies, but the unique nature of each study and the perspectives of varied stakeholders also require unique data collection and analytic approaches.

The MCRC OHDC Consortium studies are specifically aimed at improving health of children facing health disparities and inequities, and together will provide valuable information and guide future policy and practice to address the oral health needs the US Surgeon General identified 20 years ago (33). When the MCRC OHDC studies are completed in 2021, they will individually and jointly support NIDCR's goal of expanding the evidence base on effective ways to prevent and manage dental disease in children.
